# Genistein Inhibits Proliferation and Metastasis in Human Cervical Cancer Cells through the Focal Adhesion Kinase Signaling Pathway: A Network Pharmacology-Based In Vitro Study in HeLa Cells

**DOI:** 10.3390/molecules28041919

**Published:** 2023-02-17

**Authors:** Tingting Chen, Juan Wang, Min Li, Qingqing Wu, Shuna Cui

**Affiliations:** 1 Jiangsu Key Laboratory of Integrated Traditional Chinese and Western Medicine for Prevention and Treatment of Senile Diseases, Medical College of Yangzhou University, Jiangyang Middle Road 136, Yangzhou 225001, China; 2 Department of Gynecology and Obstetrics, Affiliated Hospital of Yangzhou University, Yangzhou 225009, China; 3 Jiangsu Co-Innovation Center for Prevention and Control of Important Animal Infectious Diseases and Zoonoses, College of Veterinary Medicine, Yangzhou 225001, China

**Keywords:** genistein, cervical cancer, metastasis, network pharmacology, RNA expression profiling

## Abstract

Previous studies have provided evidence that genistein exerts a therapeutic effect on different tumor cells. However, the mechanism of action of genistein against cervical cancer cells remains largely unknown. The aim of this study was to comprehensively decipher the anti-metastatic effect and molecular mechanism of genistein action on cervical cancer cells. We developed an integrated strategy from genotype to phenotype, combining network pharmacology and a transcriptome screening approach, to elucidate the underlying mechanism of action of genistein against human cervical cancer cells. In silico studies predicted that the focal adhesion pathway may be an important signaling cascade targeted by genistein treatment. Using RNA sequencing analysis, representative genes of the focal adhesion pathway were demonstrated to be significantly downregulated. Phenotypic studies revealed that genistein demonstrated strong anti-proliferative and anti-metastatic activity in HeLa cells. Moreover, genistein modulated this activity in a concentration-dependent manner. Genistein also inhibited both the activation and gene expression of FAK (Focal Adhesion Kinase) and paxillin. In addition, vimentin and β-catenin protein expression, and Snail and Twist gene expression, were strongly inhibited by genistein. Our findings provide strong evidence for a pleiotropic effect of genistein on cervical cancer cells, mediated through the focal adhesion pathway.

## 1. Introduction

Cervical cancer is known to be the fourth most common malignancy, leading to a substantial burden and threatening women’s health worldwide [1]. A persistent infection by human papilloma virus (HPV), especially HPV16 and 18, is critical for cervical cancer initiation and progression [2,3]. The implementation of HPV vaccines, HPV testing, and cervical cytology has notably decreased the incidence of early-stage cervical cancer [2,4,5]. However, patients with advanced cervical cancer still have unfavorable outcomes due to the high incidence of metastasis, which is still one of the main factors influencing the prognosis of patients. Moreover, the clinical application of immunotherapy or adjuvant chemotherapy approaches in advanced cervical cancer has not been as effective as once expected [6,7]. Therefore, there is an urgent need for novel therapeutic approaches.

Cervical cancer is a complicated disease involving the disruption of normal complex biological networks in the human body [8]. Upon HPV infection, several key molecular events are involved in the initiation and progression of cervical cancer, including the Toll-like receptor (TLR) pathway, the nuclear factor-kappa B (NF-κB) pathway, the Notch signal pathway, the Hippo-Yes-associated protein (YAP1) pathway [9,10,11], and the focal adhesion pathway [12]. However, drugs that act on single-molecular targets usually exert an unsuccessful effect or show strong toxic side effects in clinical practice. Considering the complexity of the pathogenesis of cancers, attention has switched from focusing on single drug targets towards a more systemic view of drug targets [13]. Network pharmacology is considered a powerful tool in deciphering the complexity of biological systems and provides a new concept for understanding the interplay of molecular networks of compounds [14]. This approach is now widely used in the study of the pharmacological effects of compounds, where it promotes new directions for efficient drug discovery.

Genistein is a well-studied natural flavone compound with a wide range of biological effects, including tyrosine kinase inhibitory, anti-inflammatory, phytoestrogen, and anti-cancer effects [15,16,17,18,19,20]. Several studies have already been published concerning genistein treatment during cervical cancer development, and the reported activities of genistein include inducing cell cycle G2-M phase arrest and apoptosis, inhibiting the action of histone deacetylases and DNA methyltransferases, and synergizing the radiation effect by cell cycle G2-M phase arrest and AKT (Protein Kinase B) activation [21,22,23,24,25,26]. Considering the range of activities identified in the above-mentioned studies, the precise molecular action of genistein against cervical cancer remains largely unknown. Therefore, in order to investigate the molecular action of genistein against cervical cancer more comprehensively, this study employed an integrated strategy that combined the network pharmacology approach and RNA-seq analysis to identify critical targets related to the genistein treatment of cervical cancer.

## 2. Results

### 2.1. Identification of Potential Genistein Targets against Human Cervical Cancer

**Figure 1:**
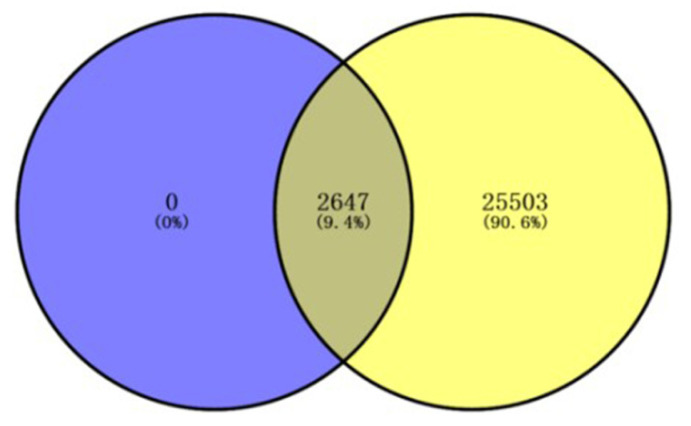
Venn plot of genes potentially involved in genistein’s action against human cervical cancer. The purple color represents the targets of genistein, and the yellow color represents the genes related to the progression of human cervical cancer.

The targets of genistein and human cervical cancer were downloaded from the Comparative Toxicogenomics Database (CTD). In total, 28,150 protein-coding genes were related to the progression of human cervical cancer, and 2647 protein-coding genes were confirmed as the effective targets of genistein. A total of 2647 genes were identified in a Venn plot (Figure 1). Since a large number of genes were identified, we performed secondary screening using an Interaction Count (human cervical cancer target > 50; genistein target > 2). Finally, 371 genes were selected as the core targets involved in the action of genistein (data not shown), and the following analysis is based on these genes.

### 2.2. PPI Network Construction and Identification of Hub Genes

**Figure 2:**
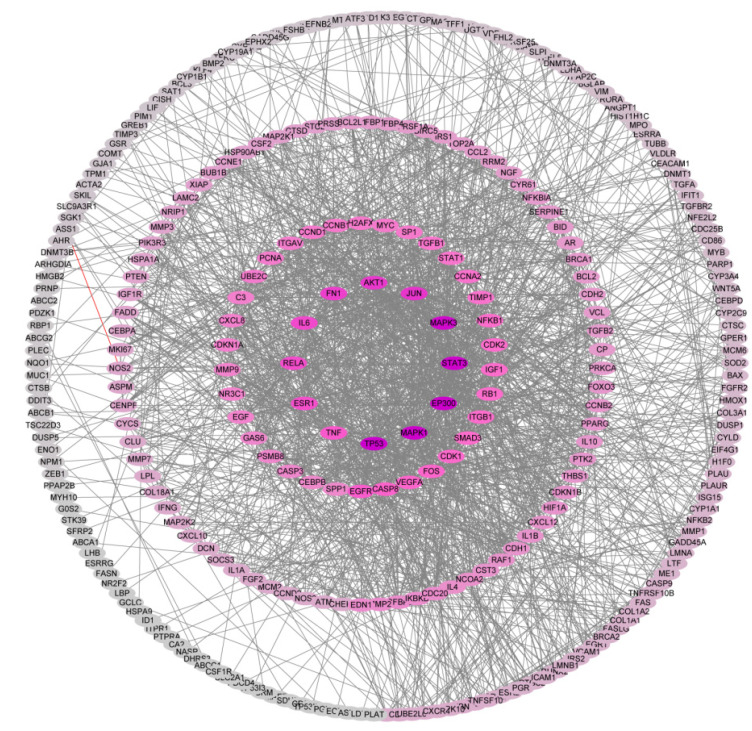
PPI network of the core genes in genistein’s action against human cervical cancer (dark purple color indicates high degree; grey color indicates low degree).

**Figure 3:**
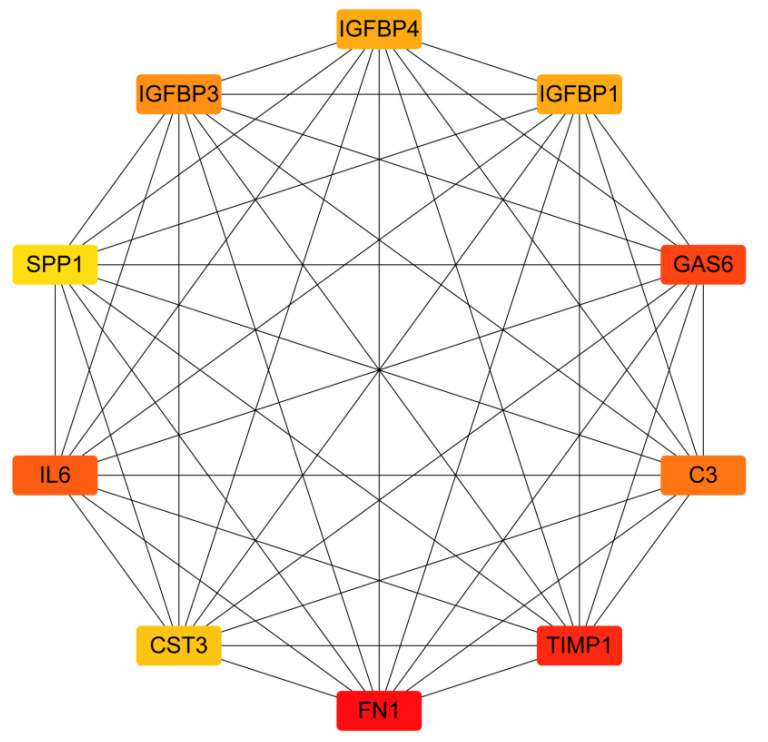
Hub gene analysis of the PPI network. The color change from yellow to red indicates the increasing importance of related genes in the network. The red color indicates the highest significance.

The 371 core targets were introduced into the STRING database to build the PPI (Protein-Protein Interaction) network of genistein’s action against human cervical cancer. A confidence score of protein and protein interaction of >0.9 was set, and data were collected and visualized using Cytoscape. As shown in Figure 2, the network contained 317 nodes and 1754 edges; the cluster coefficient was 0.33, and the network centralization value was 0.170. The top 10 hub genes in this network were subsequently identified by MCC (Maximal Clique Centrality) algometrical analysis: FN1, TIMP1, GAS6, IL6, C3, IGFBP3, IGFBP4, IGFBP1, CST3 and SPP1. The increasing significance of genes in the network is represented by the color change (yellow to red) (Figure 3).

### 2.3. GO Enrichment Analyses of Core Targets

**Figure 4:**
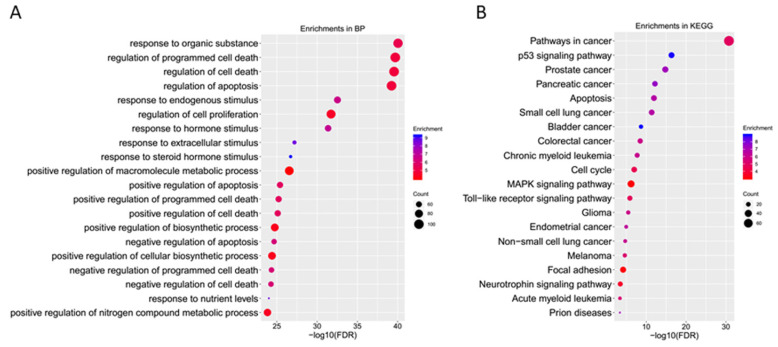
GO ontology and KEGG pathway enrichment of the core targets. (**A**) Biological processes and (**B**) KEGG pathways involved in genistein’s action against human cervical cancer.

**Figure 5:**
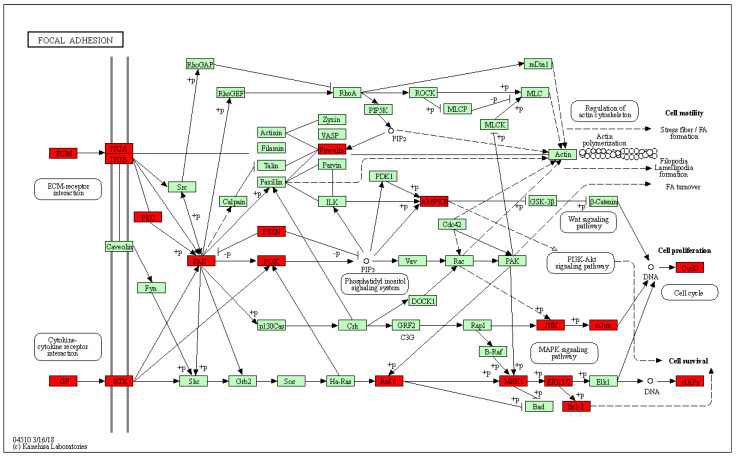
Predicted targets of genistein’s action in the focal adhesion pathway. Proteins highlighted in red indicate potential targets of genistein’s action against human cervical cancer.

The predicted core targets were further analyzed by GO (Gene Ontology) and KEGG (Kyoto Encyclopedia of Genes and Genomes) pathway enrichment in the DAVID database. The top 20 biological processes of predicted core targets were identified (Figure 4A); the core targets are mainly involved in the response to organic substances, regulation of programmed cell death, regulation of cell death, regulation of apoptosis, response to endogenous stimulus, regulation of cell proliferation, response to hormone stimulus, and response to extracellular stimulus. The enriched KEGG pathways (Figure 4B) included the following: pathways in cancer; the p53 signaling pathway; the cell cycle; apoptosis; the MAPK (Mitogen Activated Protein Kinase) signaling pathway; the TLR (Toll Like Recepter) signaling pathway; and focal adhesion. From the hub gene analysis, FN1 was selected as the most significant hub gene in genistein’s action against the cervical cancer network. Therefore, we predicted that the focal adhesion pathway is a novel pathway targeted by genistein in the treatment of cervical cancer. To gain a deeper insight, all the predicted genes in this pathway potentially regulated by genistein are highlighted in red (Figure 5).

### 2.4. Genistein Attenuates Cell Viability and Growth in HeLa Cells

**Figure 6:**
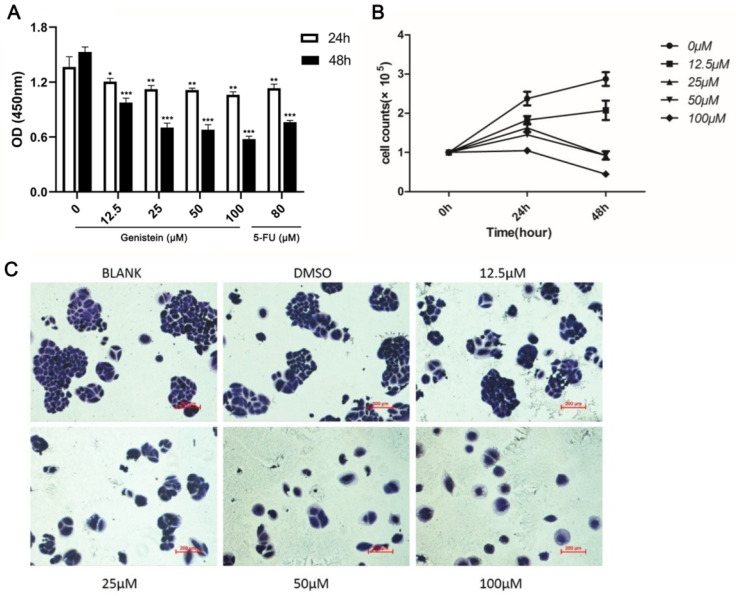
(**A**). Effects of genistein treatment on the proliferation of HeLa cells for 24–48 h were detected by CCK−8 assay. (**B**). Effects of genistein treatment on cell growth were counted by hemocytometer after 24–48 h. (**C**). Representative images of HeLa cell colony formation after genistein treatment (0–100 μM). The data shown are the average of three replicates; the experiments were performed three times independently. *** *p* < 0.001, ** *p* < 0.01, * *p* < 0.05 compared with solvent control. Scale bar: 200 μm.

According to in silico studies, genistein showed multiple effects on cervical cancer. Next, we experimentally validated these effects using human cervical cancer cells in vitro. The high−risk HPV 16 and 18 are highly associated with the pathogenesis of cervical cancer. Moreover, according to the literature, HeLa cells are more sensitive to genistein treatment. Therefore, in this study, we used HeLa cells for the following experiments [27]. As shown in Figure 6A, genistein significantly inhibited cell growth in a time- and dose-dependent manner after 24 h and 48 h of treatment by CCK−8 (Cell Counting Kit−8) assay. Moreover, 5-fluorouracil (5−FU), clinically used as an anti-tumor drug, was used as a positive control. The concentration of 5−FU was chosen based on the literature [28]. The result showed that 80 μM of 5−FU inhibited the viability of HeLa cells compared with the DMSO (Dimethyl sulfoxide) control. The results indicate that genistein exerted the same inhibitory effect on the human cervical cancer cells as the anti-cancer drug 5−FU. Moreover, we further tested the effects of genistein on cell growth by using cell number counting. The results indicate a prominent effect of genistein on cell growth; the cell number was significantly decreased after genistein (12.5–100 μM) treatment for 24–48 h (Figure 6B). Furthermore, genistein strongly inhibited the colony formation ability of cervical cells (Figure 6C). While cervical cells formed large colonies in the control group, this colony-forming ability was significantly decreased after genistein treatment, indicating that genistein (12.5–100 μM) strongly inhibited HeLa cells’ proliferation. Overall, these results demonstrate that 12.5–100 μM genistein strongly inhibited HeLa cells’ viability and proliferation.

### 2.5. Genistein Inhibits HeLa Cell Adhesion

**Figure 7:**
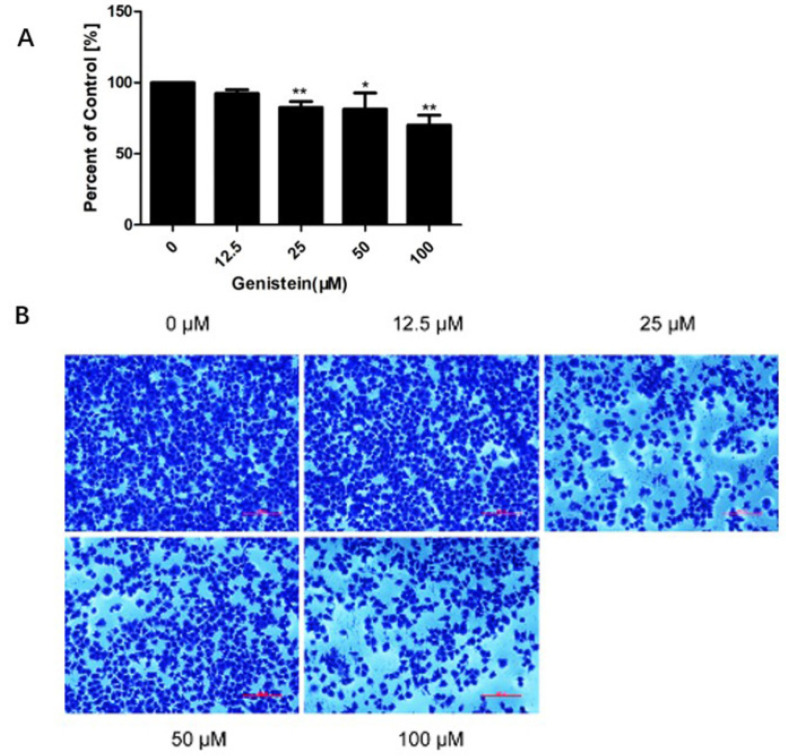
Effects of genistein on HeLa cell adhesion. Cells were grown in the presence of different doses of genistein for 24 h, and reseeded for 3 h. Adherent cells were then fixed by PFA (Paraformaldehyde) and stained with crystal violet solution. Absorbance readings were detected at OD570 nm by microplate reader. (**A**). Percentage of adhesion was then calculated based on the OD value of the adhered cells in the genistein-treated group (compared to control values (100%)). The experiment was performed three times independently, and data shown are the average of all three replicates. (**B**). Representative images from the three independent experiments. ** *p* < 0.01 vs. DMSO control; * *p* < 0.05 vs. DMSO control. Scale bar: 200 μm.

Since adhesion is the first critical step in cancer metastasis, we initially investigated the influence of genistein on HeLa cells’ adhesion. The data demonstrate that genistein dose-dependently inhibited cell adhesion in cervical cancer cells (Figure 7A,B). Compared to the control, 92%, 82%, 81%, and 71% inhibition of adhesion was observed with 12.5 μM, 25 μM, 50 μM, and 100 μM of genistein, respectively. Thus, HeLa cell adhesion was significantly inhibited after genistein (25–100 μM) treatment (*p* < 0.01; *p* < 0.05) (Figure 7A).

### 2.6. Genistein’s Inhibition of Cell Migration in the Wound−Healing Assay

**Figure 8:**
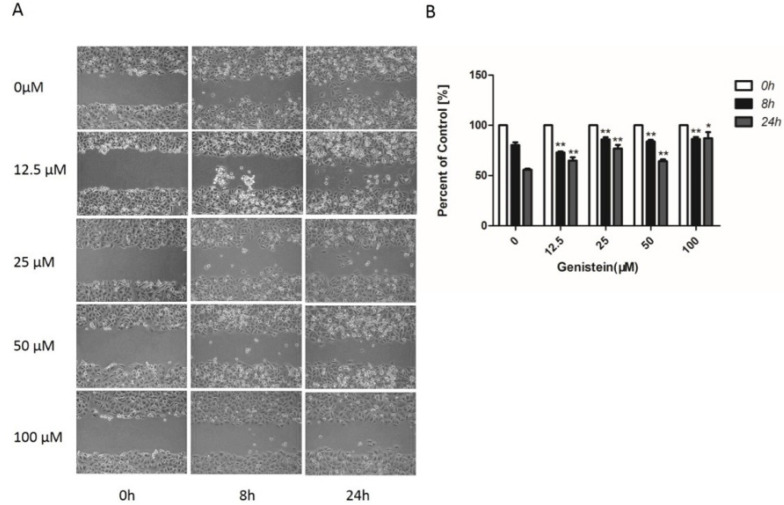
Effect of genistein on the mobility of HeLa cells. (**A**) Representative images of wound-healing assay. (**B**) Cell migration was calculated by measuring the distances from the wound edges in each treatment group using Image J software. Cell migration activity (as a percentage) was calculated from the migration distances in the genistein treatment group (compared to the control (100%)). The experiment was repeated three times. ** *p* < 0.01 vs. DMSO control. * *p* < 0.05 vs. DMSO control; Scale bar: 200 μm.

We also assessed cell migration activities using a wound-healing assay. As shown in Figure 8A,B, the continuous migration of HeLa cells was observed in the control group after 8 h and 24 h. HeLa cell migration was significantly reduced in the presence of 12.5–100 μM genistein. The results indicate that genistein strongly inhibits cell migration in a concentration-dependent manner (*p* < 0.01; *p* < 0.05).

### 2.7. Genistein Inhibited Cell Migration and Invasion of HeLa Cells in Transwell^®^ Assays

**Figure 9:**
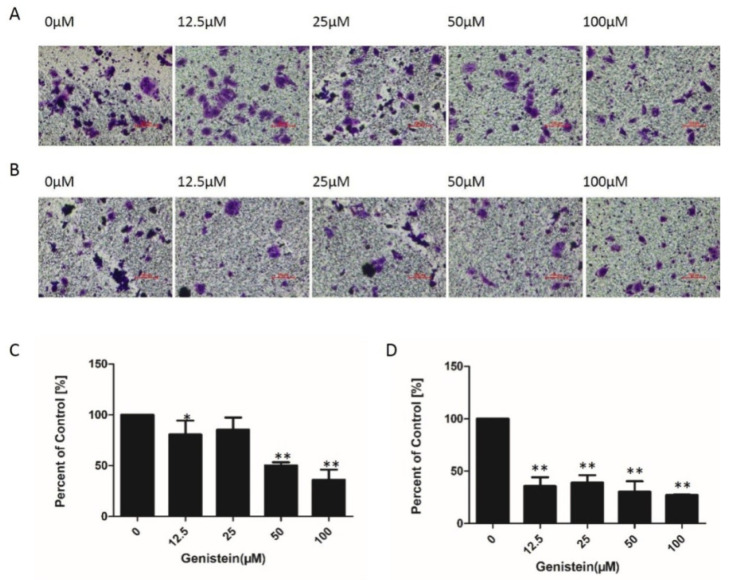
Effect of genistein on the migration and invasion activities of HeLa cells. Cells were seeded on membranes and co-cultured with different doses of genistein for 24 h. (**A**) Cells that migrated to the lower surface of the filter were fixed and stained with crystal violet, and then photographed by an inverted microscope at ×100. (**B**) Cell invasion was assessed by following cell movement through the Matrigel to the lower surface of the filter. Invading cells were fixed, stained with crystal violet, and photographed under an inverted microscope at ×100. (**C**) Cell migration was quantified from at least three images randomly using Image J software. (**D**) Cell invasion was quantified from at least three images randomly using the Image J software. The experiment was repeated three times. ** *p* < 0.01 vs. DMSO control. * *p* < 0.05 vs. DMSO control; scale bar: 200 μm.

The inhibition of cell migration by genistein was verified using the Transwell assay. A remarkable decrease in cell migration activity was observed with increasing concentrations of genistein; 12.5 μM–100 μM of genistein decreased cell migration activity by 81%, 85%, 50%, and 36% compared with the solvent control, respectively (Figure 9A–C). As shown in Figure 9B–D, genistein (12.5–100 μM) also significantly inhibited cell-invasive activity; 12.5 μM, 25 μM, 50 μM, and 100 μM of genistein decreased cell invasion activity compared with the solvent control by 36%, 39%, 30%, and 27%, respectively. Compared with the inhibition of cell migration activity, genistein exerted a stronger inhibitory effect on cell invasion activity. Moreover, compared with the cell viability results, the inhibition of cell migration and invasion was not entirely due to the inhibition of cell viability.

### 2.8. Identification of Differentially Expressed Genes (DEGs) Associated with Genistein Treatment

To further decipher the mechanism of genistein’s action on cervical cancer cells, we analyzed global gene expression profiles in genistein- and DMSO-treated cells by using RNA sequencing. Approximately 59–67 million (M) clean reads from six samples (three for genistein treatment; three for control) were obtained after deletion of the low-quality and adaptor sequences; Q30 bases ranged from 92.72% to 93.32% (data not shown). These results demonstrate that the samples were of good quality, and that the coverage of the cervical cancer cell genome was high.

**Figure 10:**
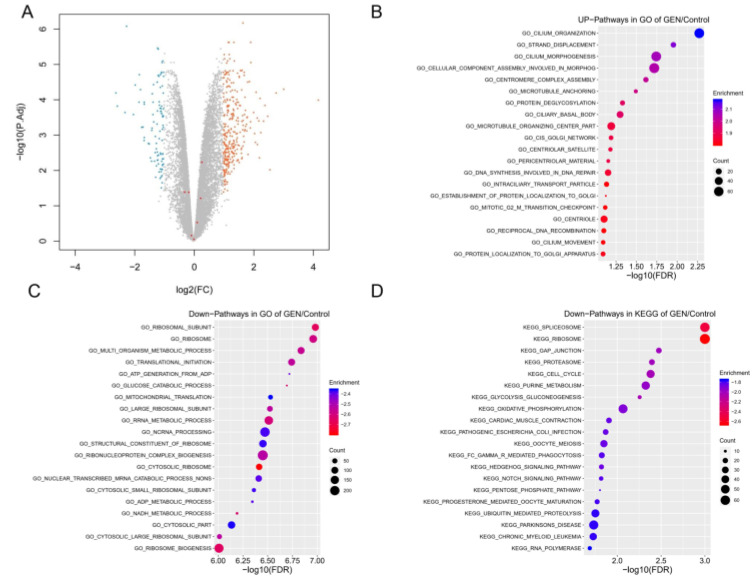
(**A**) Volcano plot of DEGs identified following genistein treatment (compared with control). Grey dots, genes with no significant difference in expression; blue dots, downregulated genes; and orange dots, upregulated genes. Fold-change was calculated using gene-normalized expression of the genistein group/gene-normalized expression of the control group. Differences in expression with a *p* value < 0.05 and a Log_2_ (fold change) > 1 were considered statistically significant. (**B**,**C**) Gene ontology enrichment of up- and downregulated DEGs after genistein treatment. (**D**) KEGG enrichment of upregulated DEGs after genistein treatment.

We previously observed significant changes in the proliferation and metastasis of cervical cancer cells after genistein treatment. The transcriptome screening results provide strong evidence that these changes were accompanied by significant differences in gene expression. A total list of expressed genes was determined using RNA-Seq data (Figure 10). Among these, genes that were upregulated (orange) and downregulated (blue) following genistein treatment (compared with control) were identified (*p* < 0.05; |log_2_FoldChange| > 1).

**Figure 11:**
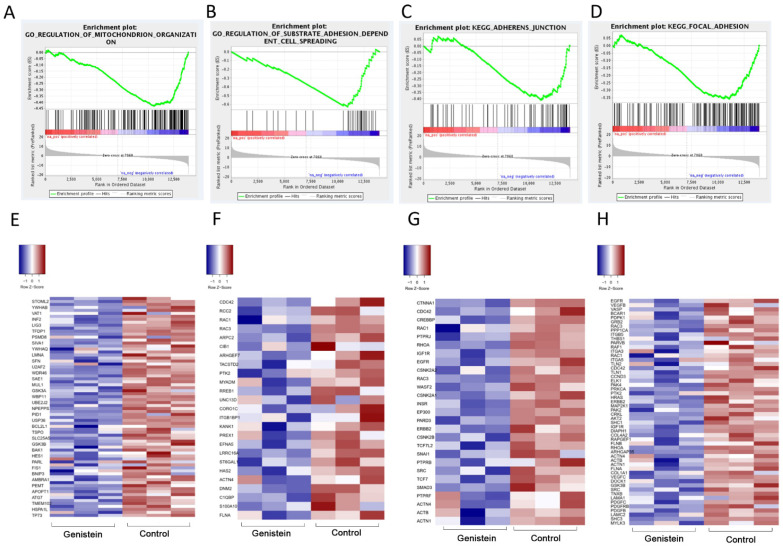
(**A**−**D**) Representative enrichment of gene signatures in genistein and control group by gene set enrichment analysis (GESA). Representative enriched gene sets are shown (FDR q value < 0.05). (**E**−**H**). Heatmap of the representative DEGs between genistein and control group in parallel with GESA analysis.

Gene ontology of the DEGs was analyzed by GESA (Gene Set Enrichment Analysis), and the upregulated and downregulated DEGs were enriched for biological process analysis. The upregulated DEGs are involved in morphological processes, including cilium organization, strand displacement, and cilium morphogenesis. In contrast, the downregulated DEGs are associated with ribosomal subunits, the ribosome, the multi-organism metabolic process, and mitochondrial translation. GESA analysis revealed that the gene clusters involved in the regulation of mitochondrion organization, substrate adhesion-dependent cell spreading, and focal adhesion were significantly downregulated (Figure 10B,C and Figure 11A,B,E,F). Moreover, the top KEGG pathways are listed in Figure 10D. The downregulated DEGs are involved in the spliceosome, ribosome, GAP junction, proteasome, cell cycle, purine metabolism, glycolysis metabolism, and oxidative phosphorylation. Previously, we predicted that the focal adhesion pathway was a promising target for genistein treatment in silico (Figure 3 and Figure 4). The RNA sequencing results validated these predictions, and numerous genes in the focal adhesion pathway were downregulated after genistein treatment, including FAK, PAK, Src, Shc, Actinin, Talin, and ILK. Moreover, mitochondrion function was also significantly inhibited by genistein, while adhesion-related pathways involving adherent junctions were also downregulated (Figure 11C,D,G,H). Interestingly, our data provide evidence that genistein mainly exerted a downregulation effect on cervical cancer cells.

### 2.9. Genistein Inhibits Activation of the FAK–Paxillin Pathway

**Figure 12:**
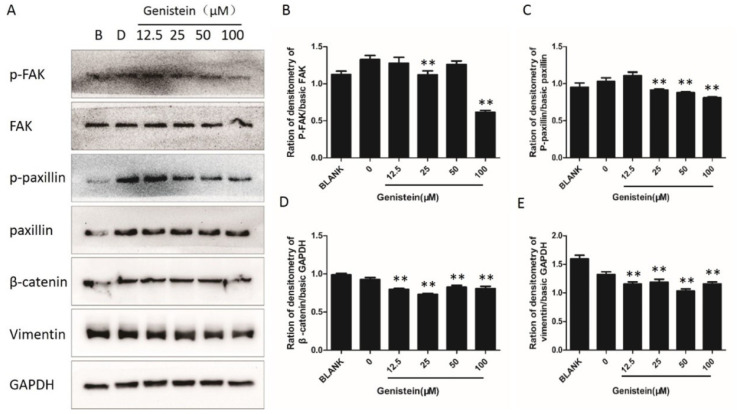
Effect of genistein on the focal adhesion protein expression in HeLa cells. (**A**) Cells were grown with or without different doses of genistein for 30 min. The expression levels of specific proteins were detected by Western blot analysis. GAPDH was used as a control. All first antibodies were used at a dilution of 1:1000. Secondary antibodies were used at a concentration of 1:3000. (**B**–**E**) Integrated optical intensity of the bands was determined by Image J software (https://imagej.net, accessed on 3 January 2023). The experiment was repeated three times. ** *p* < 0.01 vs. DMSO control.

To investigate whether genistein inhibited cell migration and invasion through inhibition of the FAK−paxillin pathway, we performed Western blot analyses to detect the expression of the relevant proteins. As shown in Figure 12, genistein treatment strongly decreased the phosphorylation of paxillin and FAK. In addition, the expression of β-catenin and vimentin was inhibited by genistein. The results suggest that the molecular mechanism of genistein on cell proliferation and metastasis involves inactivation of the FAK−paxillin pathway.

### 2.10. Genistein Inhibits Gene Expression of FAK, Paxillin, Snail, and Twist

**Figure 13:**
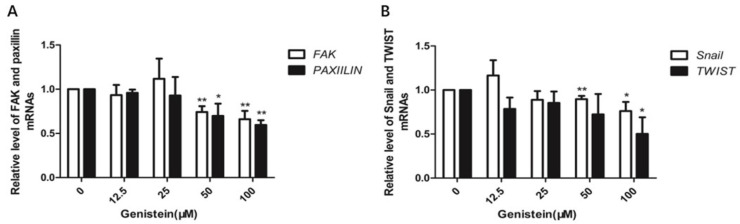
Effect of genistein on specific gene expression in HeLa cells. (**A**) Genistein inhibited FAK and paxillin, and (**B**) Snail and Twist mRNA expression in HeLa cells. Gene expression was analyzed by qRT-PCR. mRNA relative expression levels were evaluated using the 2^−△△Ct^ method. GAPDH was used as an internal control. The experiment was repeated three times. ** *p* < 0.01 vs. DMSO control; * *p* < 0.05 vs. DMSO control.

After confirming that genistein inhibits the activation of the focal adhesion pathway, we next investigated whether genistein inhibits FAK and paxillin gene expression levels. qRT-PCR results indicated that 50–100 μM genistein strongly downregulated paxillin and FAK mRNA expression. In addition, we analyzed the expression of other genes of interest. Snail is a transcription factor that regulates the expression of E-cadherin. Twist is closely associated with cervical cancer progression [29,30,31]. Our results showed that high doses of genistein (50–100 μM) inhibited Snail and Twist expression (*p* < 0.01; *p* < 0.05) (Figure 13). These results confirmed that genistein inhibits migration and invasion via Snail- and Twist-mediated EMT (Epithelial Mesenchymal Transition).

## 3. Discussion

The effects of genistein (alone or in combination with anti-cancer drugs) on growth regulation, angiogenesis, and metastasis have been extensively investigated in several different tumors [32,33,34]. Multiple molecular mechanisms have been implicated in its actions, including inhibition of the NF-κB, Wingless, and integration 1 beta-catenin (Wnt/beta-catenin); mitogen-activated protein kinase (MAPK); and phosphoinositide 3 kinase/Akt (PI3K/Akt) signaling pathways [35]. However, evidence regarding the effects of genistein on cervical cancer is limited. Therefore, the underlying mechanism of genistein’s action against human cervical cancer, especially its anti-metastatic potential, remains unknown. The present study was designed to elucidate the mechanism of genistein’s action against cervical cancer.

We first used a network pharmacological approach to predict potential targets of genistein’s action against human cervical cancer in silico. We identified 10 hub targets of genistein treatment: FN1; TIMP1, GAS6, IL6, C3, IGFBP3, IGFBP4, IGFBP1, CST3, and SPP1. FN1 encodes fibronectin, which monitors proliferation and metastasis by regulating the FAK signaling pathway in cervical cancer cells [12]. Previous in silico studies have identified the focal adhesion pathway as a key pathway in cervical cancer [36]. Moreover, the focal adhesion pathway is already considered a potential target in the treatment of highly invasive cancers [37,38,39]. Focal adhesion kinase (FAK) is an intracellular tyrosine kinase and plays an important role in the regulation of ECM integrin signaling [40,41,42,43]. Evidence is accumulating that demonstrates that FAK promotes tumorigenesis through a wide range of cellular processes, including proliferation, survival, metastasis, angiogenesis, epithelial–mesenchymal transition (EMT), cancer stem cell activities, and the metabolism of glucose, lipids, and glutamine [12,44,45]. In our in silico study, focal adhesion was identified using KEGG pathway enrichment analysis as one of the targeted pathways that regulate the anti-cancer effects of genistein (Figure 4 and Figure 11). Considering all the evidence, the FAK pathway was selected as the main pathway for experimental validation.

Phenotypic studies revealed that genistein decreased the proliferation of HeLa cells. This observation is consistent with a previous study indicating that genistein demonstrates cytotoxic properties in numerous different cell types [17,20]. Moreover, genistein is reported to inhibit the proliferation of HeLa, CaSki, and C33 cell lines, and HeLa cells are more sensitive to genistein [27]; therefore, we used the HeLa cell line for our mechanism study. According to our results, genistein also dose-dependently inhibited the HeLa cells’ adhesion and metastasis. The inhibition of adhesion is consistent with genistein’s inhibition of focal adhesion pathway activation and genistein’s inhibition of vimentin and β-catenin expression. Thus, our results provide evidence that genistein suppresses cervical cancer cell metastasis by regulating the FAK/paxillin pathway. Genistein has previously been shown to influence cervical cancer by altering epigenetic modulatory signatures and inducing apoptosis [21,23]. Our results provide strong evidence that genistein inhibits both the activation of the FAK/paxillin pathway and FAK and paxillin gene expression in cervical cancer cells. These results are consistent with the published data and our previously obtained data [17]. We showed that genistein inhibited p-FAK after only 10 min treatment and had a stable inhibitory effect after 24 h treatment in melanoma cells. In human cervical cells, genistein exerts the same behavior. Furthermore, we have previously reported that high concentrations of genistein strongly decrease Snail expression in melanoma cells [17]. Snail is an important transcription factor regulating the process of EMT; the overexpression of Snail in several tumor tissues is associated with metastasis and recurrence [46]. Here, we show that genistein also inhibits Snail expression in human cervical cancer cells.

The role of genistein in cervical cancer was also explored by RNA expression profiling. Thus, the influence of genistein on the regulation of RNA transcription, processing, and splicing in cervical cancer cells was elucidated. The top KEGG pathways enriched using the downregulated DEGs included the spliceosome, ribosome, cell cycle, RNA transport, ribosome biogenesis in eukaryotes, ubiquitin-mediated proteolysis, RNA polymerase, steroid biosynthesis, viral carcinogenesis, central carbon metabolism in cancer, microRNAs in cancer, focal adhesion, cellular senescence, and thyroid hormone signaling pathways. Using RNA sequencing analysis, we were able to confirm our in silico predictions that focal adhesion was a target pathway. In addition to inhibiting focal adhesion, genistein downregulated the expression of PKC, Src, Shc, Pak, Actinin, Talin, and ILK (Figure 11). Protein kinase C (PKC) is a serine-threonine kinase found in most cell types, where it exerts a strong influence on signal transduction events. Elevated expression of PKC has been highly associated with several human cancers, and inhibition of PKC signaling retards the growth and invasion of cervical cells [47]. The proto-oncogene tyrosine-protein kinase Src (Src) is involved in cell growth, differentiation, migration, and survival. FAK and Src are recruited upon integrin activation to form the FAK–Src complex, and this complex phosphorylates downstream adaptor proteins such as paxillin. The activated FAK–Src complex has an essential role in controlling cell shape and cell motility [48,49].

Our RNA profiling analysis revealed that the downregulated DEGs are involved in the regulation of the cell cycle. This result is consistent with our earlier published data. We previously found that genistein could block the cell cycle arrest of macrophages in the G2/M phase [50]. In addition, genistein strongly downregulated mitochondrial organization and disrupted several metabolic pathways, including the glucose catabolic process, ATP (Adenosinetriphosphate) generation from ADP (Adenosinediphosphate), and multi-organism metabolic processes. According to previous reports, genistein can trigger anti-cancer activity against several cancer cells involved in mitochondrial apoptosis [51,52,53]. However, in cervical cancer, genistein’s action on mitochondrial function has not yet been reported. Therefore, this result may herald a new direction in cervical cancer research.

In conclusion, our study provides evidence that the mode of action of genistein against cervical cancer is comprehensive, from genotype to phenotype. Our results reveal that genistein exerts its anti-proliferation and anti-metastatic activities against cervical cancer by interacting with several key pathways. Genistein inhibits the FAK/paxillin pathway and strongly regulates Twist/Snail-mediated EMT, two pathways related to the progression of cervical cancer. Differing from the dual function of genistein in regulating the metastasis of melanoma cells [17], the predominant inhibitory effects of genistein on cervical cancer cells were identified based on RNA expression profiling and phenotypic studies. The novel mechanisms of action of genistein against cervical cancer identified in this study should prove useful in future research and in clinical applications. Overall, our study provides strong evidence that genistein is a promising chemotherapeutic agent against cervical cancer.

## 4. Materials and Methods

### 4.1. Prediction of Genistein’s Anti-Human Cervical Cancer Targets

All verified targets of genistein and human cervical cancer were screened in the Comparative Toxicogenomics Database (CTD) (http://ctdbase.org/) (accessed on 13 June 2018) [54,55,56,57]. CTD is a unique tool in which three types of core data can be obtained, including chemical–gene (and protein) interactions, chemical–disease relationships, and gene–disease relationships. It offers the basis for testable hypotheses about the mechanisms underlying the etiology of environmental diseases [54]. The selected genes were further selected according to the interaction count value, compound targets ≥ 2, disease targets ≥ 50. Finally, potential targets of genistein against human cervical carcinomas were obtained by overlapping the compound targets and disease targets.

### 4.2. PPI Network Analysis and Identification of Hub Genes

The STRING database [58] was applied to build the PPI network. The confidence score for protein–protein interactions was selected as >0.9. The PPI network for genistein against human cervical carcinoma was visualized using Cytoscape (v3.7.1). The hub genes in the PPI network were calculated using the CytoHubba plugin based on the MCC algorithm [59].

### 4.3. GO and KEGG Pathway Enrichment Analyses of Core Targets

The Database for Annotation, Visualization, and Integrated Discovery (DAVID) (https://david.ncifcrf.gov/) (accessed on 17 September 2018) was used for Gene Ontology and KEGG pathway enrichment analysis of core targets. Bubble charts of BP and KEGG pathways were produced by ggplot2, implemented in the R platform.

### 4.4. Experimental Verification

#### 4.4.1. Reagent Source

Fetal bovine serum (FBS), 100× penicillin, streptomycin, and Dulbecco’s modified Eagle’s medium (DMEM) were purchased from Lonza (Verviers, Belgium). Genistein was obtained from Selleck (Shanghai, China). Genistein was dissolved in DMSO with a stock solution of 100 mM and diluted in culture medium to final concentrations (12.5–100 µM). Phosphatase and protease inhibitor cocktail set I and WST-8 buffer were purchased from Biotool (Shanghai, China). Enhanced chemiluminescence (ECL) detection buffer was purchased from Beyotime Biotechnology (Shanghai, China). Antibodies used in this study were purchased from Cell Signaling Technology (Danvers, MA, USA), and are listed in Table 1.

#### 4.4.2. Cell Culture and CCK-8 Cell Viability Test

The human cervical cancer cell line HeLa (HPV 18 positive) was purchased from the Type Culture Collection of the Chinese Academy of Sciences (Shanghai, China). Cells were cultured in DMEM with 10% FBS, penicillin (100 units/mL), and streptomycin (100 µg/mL), in an incubator at 37 °C with 5% CO_2_. The CCK-8 test was performed as previously described [17,41]. Briefly, HeLa cells (1 × 10^5^ cells/mL) were cultured in DMEM with different doses of genistein and solvent control for 24–48 h. CCK-8 was then added to the culture medium and was incubated for 1 h. The absorbance was measured at a wavelength of 450 nm (OD450 nm) in a microtiter plate reader (BioRad, Hercules, CA, USA).

#### 4.4.3. Colony Formation Assay

Cells (1 × 10^3^ cells/well) were initially grown in DMEM with or without different concentrations of genistein and solvent control. After 7–10 days, the cells were fixed with PFA and stained with crystal violet. Images of five randomly selected fields were captured under an inverted microscope (Nikon, Tochigi, Japan).

#### 4.4.4. Adhesion Assay

This assay was performed according to previous protocols [60,61]. Cells (1 × 10^5^ cells/mL) were grown for 2 h and then cultured with or without genistein (0–100 µM) for another 24 h in a 12-well plate. Cells were collected and reseeded with the concentration of 1 × 10^5^ cells/mL in a 96-well plate for 3 h. Afterwards, cells were washed, fixed with PFA, and finally stained with crystal violet. Adhesion was assessed at OD570 nm using a microplate reader. Images of five randomly selected fields were captured under an inverted microscope. The percentage of adherent cells was calculated from the OD values of the genistein-treated group (relative to the OD values of the control group).

#### 4.4.5. Wound-Healing Mobility Assay

This assay was performed according to previous protocols [17]. Briefly, cells (1 × 10^5^ cells/well) were grown in a 6-well plate for 24 h to achieve 90% confluency. The medium was discarded and cell monolayers were scratched with a sterile P200 micropipette tip. After the debris was washed away, cells were allowed to grow in serum-free medium with different doses of genistein and solvent control for 24 h. Images of the wound areas were captured at 0 h, 8 h, and 24 h. Cell migration was calculated by measuring cell distances from the wound edges in each treatment group. Cell migration (as a percentage) was calculated based on the migration distances in the genistein-treated group (relative to those of the control group).

#### 4.4.6. Transwell Assay

The assay was performed according to previous protocols [17]. Cells were cultured in a Transwell^®^ cell culture chamber (8 mm pore size; Corning, Lowell, MA, USA) at a density of 2 × 10^4^/well. For a 24-h invasion assay, the chambers were first coated with Matrigel^TM^. A cell suspension in serum-free DMEM was cultured in the upper chamber of the Transwell^®^ insert with or without different doses of genistein and solvent control. The lower part was filled with DMEM containing 20% FBS as a chemoattractant. After 24 h, cells that migrated or invaded into the lower surface of the membrane were fixed with 4% PFA and stained with crystal violet solution. Images of five random fields were captured by an inverted microscope. Cell migration/invasion was subsequently assessed using Image J software. Cell migration/invasion was calculated (as a percentage) from the relative numbers of cells in the genistein-treated and control groups.

#### 4.4.7. Western Blotting Assay

The assay was carried out according to previous protocols [17]. Briefly, cells were grown with or without different concentrations of genistein (12.5–100 μM) and solvent control for 30 min. Cells were then harvested and lysed in ice-cold cell lysis buffer. After protein extraction, the total protein concentration was quantified using standard protocols. Total protein (20 μg of protein/lane) was then separated on a 10% SDS-PAGE gel by electrophoresis. Separated proteins were transferred onto PVDF (Polyvinylidene Fluoride) membranes (BioRad, USA) and subsequently probed with the respective primary antibody followed by an HRP (Horse Radish peroxidase) -conjugated secondary antibody. Finally, the blots were developed by ECL (Electrochemiluminescence).

#### 4.4.8. RNA Sequencing

RNA sequencing was carried out as previously reported [62,63]. Cells were grown with DMSO and genistein (50 μM) for 5 h. Total RNA was isolated using the RNeasy Plus Mini Kit, following the manufacturer’s instructions (Qiagen, Germantown, MD, USA). The quality of the RNA was first confirmed, and then the RNA was sequenced using the Illumina Hiseq X ten platform in GeneChem. Data from the sequencer were first subjected to quality control using FastQC and trimmed using trimgalore. Data processing included (i) trimming the Illumina adapter sequence and low-quality bases (phred score < 20) at the 3′ end; (ii) discarding the reads with a length shorter than 20 (the paired reads were removed if any of the two reads did not meet the minimum length). Duplicates were then removed using Picard. For each sample, we counted the reads of individual transcripts using htseq-count. Differential analysis between treatments (genistein) and the control was performed using a count-based method, limma, implemented in R, and voom for normalization [64,65]. Significantly expressed genes were first screened for BH-adjusted *p* values less than 0.05 [66] and further filtered using a 2-fold-change minimum boundary (up- and downregulated genes labeled in the volcano plot). In parallel, we used GSEA v3.0 (Broad Institute, PreRanked mode) for enrichment analysis. To ensure consistency in our method for identifying significant genes, we used the t-statistic output from the limma as a metric for ranking. Here, 1000 gene set permutations were set as default, and gene sets were obtained by collecting pathways from KEGG and biological processes from GO. A gene set with an FDR q value less than 0.05 was considered significantly enriched. For the heatmap, log_2_ transformed FPKM values (fragments per kilobase of transcript per million mapped reads) of the significant genes were used as input for heatmap generation.

#### 4.4.9. Quantitative Real-Time RT-PCR

Cells were grown with or without different doses of genistein and solvent control for 5 h. Total RNA was isolated using the RNeasy Plus Mini Kit. The cDNA was synthesized using a reverse transcription reagent kit (Selleck, Shanghai, China). Target genes were amplified with the following specific primers in the Light Cycler^®^ 96 Real-Time PCR System (Roche, Indianapolis, IN, USA):

GAPDH: 5′-GGAGCGAGATCCCTCCAAAAT-3′ (forward) and

5′-GGCTGTTGTCATACTTCTCATGG-3′ (reverse);

FAK: 5′-TGGTGCAATGGAGCGAGTATT-3′(forward) and

5′-CAGTGAACCTCCTCTGACCG-3′(reverse);

Paxillin: 5′-CTGCTGGAACTGAACGCTGTA-3′ (forward) and

5′-GGGGCTGTTAGTCTCTGGGA-3′ (reverse);

Snail: 5′-TCGGAAGCCTAACTACAGCGA-3′ (forward) and

5′-AGATGAGCATTGGCAGCGAG-3′ (reverse);

Twist: 5′-GTCCGCAGTCTTACGAGGAG-3′ (forward) and

5′-GCTTGAGGGTCTGAATCTTGCT-3′ (reverse).

GAPDH was used as a normalization control. Each treatment was tested in triplicate. The relative expression levels of genes were normalized using the 2^−ΔΔCt^ method.

### 4.5. Statistical Analyses

Data are reported as the mean ± S.D. from at least three independent experiments. A *p* < 0.05 was used to measure statistical significance. Student’s *t*-tests were used to compare statistical significance between the treated groups.

## Figures and Tables

**Table 1:** List of antibodies used in the experiments.

	Name	Source	ID
1	Paxillin	Rabbit, pAb	#2542 Cell signaling
2	Anti-rabbit IgG, HRP-linked antibody	Goat	#7074 Cell signaling
3	Phospho-Paxillin (Tyr118)	Rabbit, pAb	#2541 Cell signaling
4	FAK	Rabbit, pAb	#13430 Cell signaling
5	Phospho-FAK (Tyr925)	Rabbit, pAb	#9330 Cell signaling
6	Vimentin (D21H3)	Rabbit, mAb	#9782 Cell signaling
7	β-Catenin (D10A8)	Rabbit, mAb	#9782 Cell signaling
8	GAPDH-HRP	Mouse mAb	#: ab011 Multi Science

## Data Availability

The data presented in this study are available on request from the corresponding author.

## Sample Availability

Samples of the compounds are not available from the authors.
